# Spherical Pendulum Small Oscillations for Slewing Crane Motion

**DOI:** 10.1155/2014/451804

**Published:** 2014-01-09

**Authors:** Alexander V. Perig, Alexander N. Stadnik, Alexander I. Deriglazov

**Affiliations:** ^1^Manufacturing Processes and Automation Engineering Department, Engineering Automation Faculty, Donbass State Engineering Academy, Shkadinova 72, Donetsk Region, Kramatorsk 84313, Ukraine; ^2^Department of Technical Mechanics, Engineering Automation Faculty, Donbass State Engineering Academy, Shkadinova 72, Donetsk Region, Kramatorsk 84313, Ukraine

## Abstract

The present paper focuses on the Lagrange mechanics-based description of small oscillations of a spherical pendulum with a uniformly rotating suspension center. The analytical solution of the natural frequencies' problem has been derived for the case of uniform rotation of a crane boom. The payload paths have been found in the inertial reference frame fixed on earth and in the noninertial reference frame, which is connected with the rotating crane boom. The numerical amplitude-frequency characteristics of the relative payload motion have been found. The mechanical interpretation of the terms in Lagrange equations has been outlined. The analytical expression and numerical estimation for cable tension force have been proposed. The numerical computational results, which correlate very accurately with the experimental observations, have been shown.

## 1. Introduction

The solution of nonlinear differential equations for spherical pendulum swaying requires the introduction of modern numerical computational analysis techniques. Such computer techniques allow us to produce approximate solutions of the posed problems. However, the linearized models for spherical pendulum small swaying motion provide a clear mechanical explanation and the first numerical approximation of the complex spherical motion problems. This fact confirms the significance of the small oscillation problems for the modern field of lifting-and-transport machines. A series of modern research efforts [1–22] have been focused on the solution of a spherical pendulum with moving pivot center.

Abdel-Rahman and Nayfeh [1] have studied the dynamic problem for a “crane boom tip-payload” mechanical system focused on the reduction of payload lateral vibrations by reeling and unreeling the hoist cable. In this work, the authors have derived two- and three-dimensional models for a spherical pendulum taking into account the linearity of transport motion.

Adamiec-Wójcik et al. [2] have provided a numerical finite-element simulation of the supporting frame of an offshore crane portal construction. Within the proposed model, supporting frame elastic oscillations define both the payload motion and the displacements of the suspension center with a payload on the cable. The derived model also incorporates ship vibration motion.

Aston has obtained two independent motion equations for a spherical pendulum taking into account viscous friction effects. This model neglects the components of the Coriolis inertia force [3].

Betsch et al. [4] have described rotary crane dynamics in terms of redundant coordinates with an introduction of differential-algebraic equations (DAEs). The authors' approach focused on the simultaneous incorporation of a large total number of redundant coordinates and the augmentation constraints do not fully describe the particular cases of rotary crane dynamics.

Blajer and Kołodziejczyk [5] have derived governing DAE equations for the open-loop control formulation that forces the underactuated crane system to complete the partly specified motion. It is necessary to note that this paper deals primarily with controlled in-plane motions of the crane system with linear transport motion. Blajer and Kołodziejczyk [6] have investigated the control of underactuated crane mechanical systems with servo constraints. This work takes into account the restoring load gravity force in the horizontal plane. However, the derived mathematical model assumes primarily linear transport motion without the Coriolis inertia force. Blajer and Kołodziejczyk [7] have presented a conceptual research article focused on the building of n-index DAE systems for a relevant dynamic description of partly specified crane system motion. Blajer and Kołodziejczyk [8] have introduced a carefully argued computational technique for numerical simulation of cranes based on the use of dependent coordinates. The advantages of dependent coordinates' usage have been outlined. It is necessary to note that with each new research effort, the authors apply much more sophisticated computational methods and derive more exact governing dynamic equations. However, this paper is also based on the same simple pendulum model as the first approaches, wherein the Coriolis inertia force and the restoring load gravity force are ignored. Blajer et al. [9] have simulated a two-tier framed human arm model with segments of equal length. The application of this model seems to be very useful for the dynamic analysis of a swaying load in the horizontal plane during rotation of a crane boom. Blajer and Kołodziejczyk [10] have developed improved DAE equations for more precise rotary crane dynamics simulation. But, additional dimensional analysis for the explicit forms of the governing equations would greatly enhance the work.

Cha et al. [11] have applied a topological modeling approach to a dynamic simulation of multiple floating cranes in shipyards taking into account crane boom oscillations.

Ellermann et al. [12] have introduced the vector of generalized gyroscopic and Coriolis forces for a mathematical description of nonlinear dynamics of floating cranes. This work used a comprehensive model with a set of 20 differential equations but does not allow proper analysis of particular cases of floating jib crane-load system motions.

Erneux and Kalmár-Nagy [13] have proposed a plane pendulum container crane model with linear transport trolley motion. This model illustrates linear displacement of the plane pendulum suspension center.

Glossiotis and Antoniadis [14] have derived a four-degrees-of-freedom (4DOF) model for the rotary crane system, incorporating both hoisting and slewing payload motions and thus is able to handle cases where hoisting can be simultaneously applied to the rotary motion.

Ibrahim [15] has studied the issues of multiplicative noise and noise-enhanced stability of spherical pendulums.

Kortelainen and Mikkola [16] have introduced data management techniques within an approach using a semantic data model for payload oscillation problems.

Mitrev [17] has applied his generalized approach with the introduction of an inertia force vector to the case of linear transport motion of the pendulum pivot center. Grigorov and Mitrev [18] have investigated a spherical pendulum dynamic model for a numerical solution of a freely suspended swinging load problem. They have derived absolute trajectories of both the pendulum suspension center and the swinging load.

Osiński and Wojciech [19] have analyzed the hoist motion of a mechanical system consisting of a load, hoist rope, and elastic crane jib. This study mainly deals with rectilinear load oscillations and lateral crane jib vibrations.

Ren et al. [20] have used a mixed approach combining both analytical and finite-element techniques for the Lagrangian function of a mechanical system. They have determined the relations between load swaying, crane boom flexibility, and wave motions. Schaub [21] has implemented a rate-based ship-mounted crane payload pendulation control system. This research is focused on a kinematic approach to payload stabilization with an emphasis on the usage of inertial measurement unit (IMU) information.

Uchiyama [22] has applied control theory techniques for a robust crane controller design that provides robustness with respect to changes in cable length and transported payload mass without iterative computations.

At the same time, these known articles [1–22] do not do justice to load swaying descriptions using Coriolis effects in relative motion. The present paper focuses on these issues.

The present paper focuses on the Lagrange mechanics-based description of small oscillations for a spherical pendulum with a uniformly rotating suspension center.

The prime novelty statement of the present paper is based on the rational introduction of a Cartesian coordinate system for study of small relative swaying of a payload and substantiation of uniformity of crane boom rotation.

## 2. Kinematic Analysis

**Figure 1:**
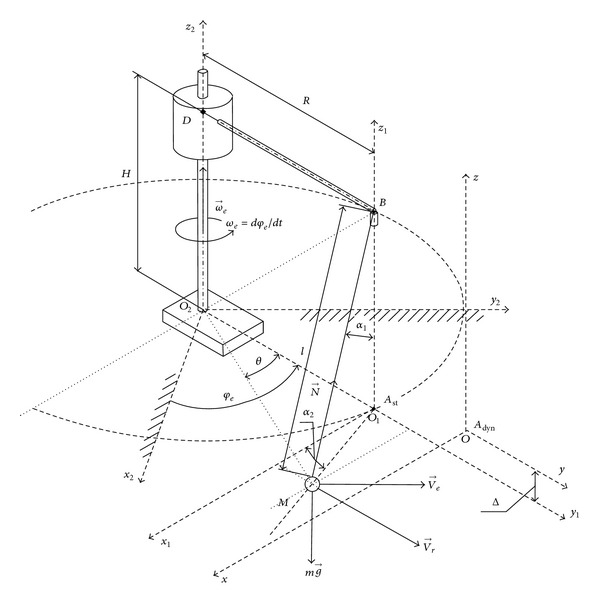
Computational spatial scheme of spherical pendulum *M*, swaying on the cable *MB* during crane boom *BO*_2_ slewing motion for the linearized model derivation in Cartesian coordinates.

In order to solve the problem, we introduce the Lagrange equations for the motion of the mechanical system “crane boom *BO*_2_-load *M*” which is shown in Figure 1.

The computational scheme in Figure 1 may be described with an introduction of three degrees of freedom. For generalized coordinates, we assume the rectangular coordinates *x*, *y*, and *z* of the load and the angle *φ*_*e*_ = *ω*_*e*_ *t* of crane boom *BO*_2_ uniform rotation in the horizontal plane (*xy*) with the constant angular velocity *ω*_*e*_ around the vertical axis *O*_2_ *z*_2_. We denote the fixed inertial coordinate system as *x*_2_ *y*_2_ *z*_2_ and the moving noninertial frame of reference as *x*_1_ *y*_1_ *z*_1_, which is rigidly bounded with the crane boom *BO*_2_. Rotation of the moving noninertial frame of reference *x*_1_ *y*_1_ *z*_1_ around the fixed inertial coordinate system *x*_2_ *y*_2_ *z*_2_ defines the transportation motion. The motion of load *M* relative to the moving noninertial frame of reference *x*_1_ *y*_1_ *z*_1_ defines the relative motion.

The scalar of the load *M* transportation velocity is defined as *V*_*e*_ = *ω*_*e*_ · *O*_2_ *M*, and transportation velocity vector **V**_*e*_ is perpendicular to **O**_2_ **M**, that is, the scalar product (**V**_*e*_, **O**_2_ **M**) = 0 (Figure 1). The relative velocity vector **V**_*r*_ of the load *M* is defined as **V**_*r*_ = (*dx*_1_/*dt*, *dy*_1_/*dt*, *dz*_1_/*dt*) in Figure 1. In the initial time *t* = 0, load *M* has the vertical *BA*_st_ in-line position; that is, the load *M* initial position coincides with the static equilibrium position *A*_st_ for the load *M* on the cable *MB*.

At time *t* = 0, the load *M* has zero absolute velocity and the initial relative velocity is *V*_*rx*_(0) = +*ω*_*e*_ · *BD* (Figure 1). In Figure 1, we denote the angle *θ*, which is the current angle *A*_st_ *O*_2_ *M*, that is, the angle between the axis *y*_1_ and the radius-vector **O**_2_ **M**, where sin(*θ*) = *x*_1_/*O*_2_ *M*; cos(*θ*) = (*R* + *y*_1_)/*O*_2_ *M*. The vertical coordinate *z*_1_ in Figure 1 is defined as *z*_1_ = *l* − *l* · cos(*α*_1_), where *α*_1_ is the angle between the cable *MB* and the vertical.

For small angles *α*_1_, the projections of the absolute velocity vector may be written as

(1) Vx1=(dx1dt)−Ve·cos(θ),Vy1=(dy1dt)+Ve·sin(θ),Vz1=(dz1dt)=(dα1dt)·l·sin(α1).

Taking into account Figure 1, the system of (1) for the projections of the absolute velocity vector takes the following form:

(2) Vx1=(dx1dt)−(dφedt)(R+y1),Vy1=(dy1dt)+(dφedt)x1,Vz1=(dz1dt).

The square of absolute payload *M* velocity may be written as *V*_abs_^2^ = *V*_*x*_1__^2^ + *V*_*y*_1__^2^ + *V*_*z*_1__^2^:

(3) Vabs2=(dx1dt)2+(dy1dt)2+(dz1dt)2 +(dφedt)2(x12+(y1+R)2) +2(dφedt)(x1(dy1dt)−(y1+R)(dx1dt))=((dx1dt)−(dφedt)(R+y1))2 +((dy1dt)+(dφedt)x1)2+(dz1dt)2.

## 3. Dynamic Analysis

The slewing motion of the mechanical system “crane boom *BO*_2_-load *M*” in Figure 1 is governed by the vector equation for the rate of change of moment of momentum **H**_3_^*O*_2_^ for the system “crane boom *BO*_2_-load *M*” with respect to point *O*_2_ in the inertial reference frame *x*_2_ *y*_2_ *z*_2_:

(4)   dH3O2(x2y2z2)dt=∑MO2.

The vector equation (4) contains the following components:

(5) $$\begin{array}{c} H_{3}^{O_{2}}=I_{33}^{O_{2}}\omega_{e}, \end{array}$$

(6) $$\begin{array}{c} I_{33}^{O_{2}}={\left(I_{33}^{O_{2}}\right)}_{BO_{2}}+m\left(x_{1}^{2}+{\left(R+y_{1}\right)}^{2}\right), \end{array}$$

(7) $$\begin{array}{c} \sum M_{}^{O_{2}}=M_{DT}^{O_{2}}-M_{FT}^{O_{2}}=\left(M_{DT}^{O_{2}}-M_{FT}^{O_{2}}\right)k, \end{array}$$

where (*I*_33_^*O*_2_^)_*BO*_2__ is the element of mass moment of inertia for the crane boom *BO*_2_ in inertial fixed on earth reference frame *x*_2_ *y*_2_ *z*_2_ with respect to unit vector **k** and *m*(*x*_1_^2^ + (*R*+*y*_1_)^2^) is the element of mass moment of inertia for the payload *M* in inertial fixed on earth reference frame *x*_2_ *y*_2_ *z*_2_ with respect to unit vector **k**.

The external moment of gravitational force **M** ^*O*_2_^(**m** **g**) = 0 in (4) and (7) because **m** **g**↑↓**k**.

For the system “crane boom-payload,” the cable reaction force **N** is the internal force. So in (4) and (7), we have **M** ^*O*_2_^(**N**) = 0.

Substitution of (5), (6), and (7) into (4) yields the following scalar equation for the rate of change of moment of momentum *H*_3_^*O*_2_^ for the system “crane boom *BO*_2_-load *M*” with respect to point *O*_2_ in the inertial reference frame *x*_2_ *y*_2_ *z*_2_:

(8) ddt(((I33O2)BO2+m(x12+(R+y1)2))(dφedt))  =MDTO2−MFTO2,

where driving *M*_*DT*_^*O*_2_^ and frictional *M*_*FT*_^*O*_2_^ torques are the technically defined functions for specific electric drive systems.

## 4. Nonlinear Mathematical Model with Lagrange Equations' Introduction

Taking into account (3) for the square of absolute payload *M* velocity *V*_abs_^2^, and by adding the kinetic energy for a slewing crane boom according to (6), we will have the following expression for “crane boom-payload” kinetic energy:

(9) T=m2(((dx1dt)−(dφedt)(R+y1))2+((dy1dt)+(dφedt)x1)2+(dz1dt)2)+I33O22(dφedt)2.

For further analysis, we will assume that the crane boom slewing angular velocity is the constant *dφ*_*e*_/*dt* = *ω*_*e*_. We will use the derived expression for kinetic energy for the left-hand sides of Lagrange equations in the noninertial reference frame *x*_1_ *y*_1_ *z*_1_:

(10) $$\begin{array}{c} \frac{d}{dt}\left(\frac{\partial T}{\partial {\dot{x}}_{1}}\right)-\frac{\partial T}{\partial x_{1}}=Q_{x_{1}},\\ \frac{d}{dt}\left(\frac{\partial T}{\partial {\dot{y}}_{1}}\right)-\frac{\partial T}{\partial y_{1}}=Q_{y_{1}},\\ \frac{d}{dt}\left(\frac{\partial T}{\partial {\dot{z}}_{1}}\right)-\frac{\partial T}{\partial z_{1}}=Q_{z_{1}}. \end{array}$$

Taking into account the nonlinearity and nonconservatism of the cable reaction force **N**, we have the following formulae for the generalized forces in the noninertial reference frame *x*_1_ *y*_1_ *z*_1_:

(11) Qx1=−N(x1l),Qy1=−N(y1l),Qz1=+N(l−z1l)−mg.

Taking into account (9), (11), the equations (10) in the noninertial reference frame *x*_1_ *y*_1_ *z*_1_ will finally take the following form:

(12) m(d2x1dt2)−m(dφedt)2x1−m(d2φedt2)(R+y1) −2m(dφedt)(dy1dt)=−N(x1l),m(d2y1dt2)−m(dφedt)2(R+y1)+m(d2φedt2)x1 +2m(dφedt)(dx1dt)=−N(y1l),m(d2z1dt2)=−mg+N(l−z1l),l2=x12+y12+(z1−l)2.

**Figure 2:**
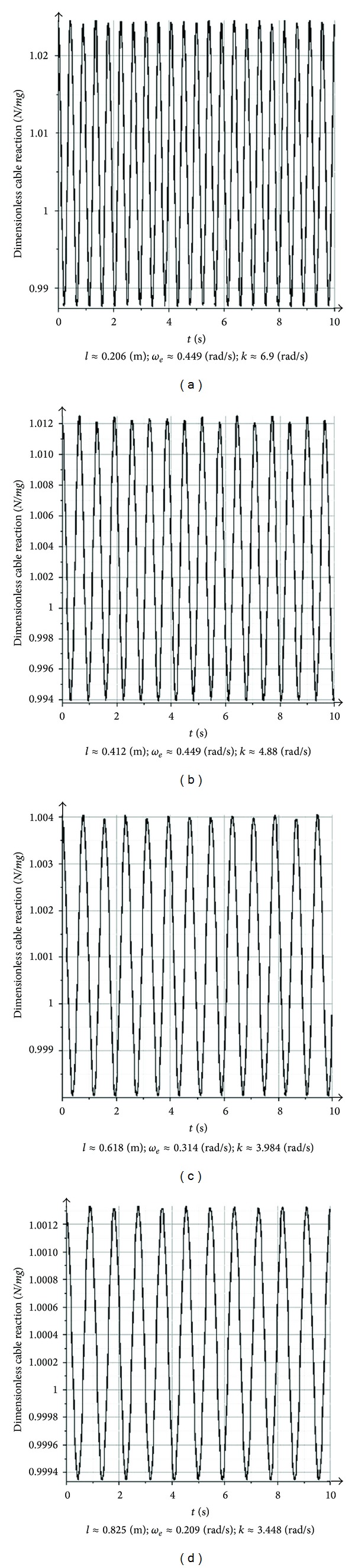
Time dependencies of dimensionless cable reactions *N*/*mg* in the first approximation for fixed cable lengths *l* = 0.206 m (a); *l* = 0.412 m (b); *l* = 0.618 m (c); and *l* = 0.825 m (d).

After two times differentiation of forth equations in system (12), we will have the following formula for the cable reaction force *N* (Figure 2) from third equation of (12):

(13) N=((ml[((x1(d2x1dt2)+(dx1dt)2+y1(d2y1dt2)+(dy1dt)2)×(l2−x12−y12)+(x1(dx1dt)+y1(dy1dt))2)])×(l2−x12−y12)−2)+mgl(l2−x12−y12).

So with an introduction of (13) to the first and second equations of system (12), we will have the explicit nonlinear ODE system for payload *M* swaying in Cartesian coordinates.

## 5. Linearized Mathematical Model

Further for the small angle *α*_1_, we may assume that *z*_1_ and *d* ^2^ *z*_1_/*dt* ^2^ are the small parameters. So the nonlinear equation (13) yields

(14) $$\begin{array}{c} N\approx mg. \end{array}$$

Then, taking into account (14), the linearized system (12) for the two variables *x*_1_ and *y*_1_ takes the following form:

(15) $$\begin{array}{c} \frac{d^{2}x_{1}}{dt^{2}}-{\left(\frac{d\varphi_{e}}{dt}\right)}^{2}x_{1}-2\left(\frac{d\varphi_{e}}{dt}\right)\left(\frac{dy_{1}}{dt}\right)=-g\left(\frac{x_{1}}{l}\right),\\ \frac{d^{2}y_{1}}{dt^{2}}-{\left(\frac{d\varphi_{e}}{dt}\right)}^{2}\left(R+y_{1}\right)+2\left(\frac{d\varphi_{e}}{dt}\right)\left(\frac{dx_{1}}{dt}\right)=-g\left(\frac{y_{1}}{l}\right). \end{array}$$

**Figure 3:**
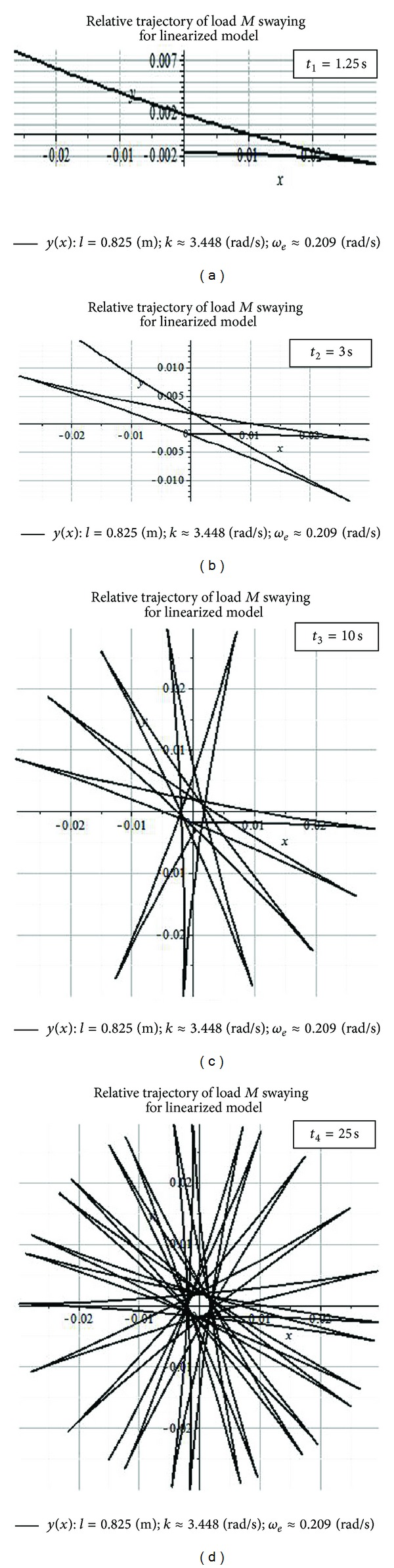
The relative trajectories for load *M* on the cable during uniform rotational motion of the crane boom *BO*_2_ for the moments of time *t*_1_ = 1.25 s (a), *t*_2_ = 3 s (b), *t*_3_ = 10 s (c), and *t*_4_ = 25* *s (d).

So we derive the system (15) of differential equations for relative motion of load *M* on cable *BM*  with a movable suspension center *B*, which is fixed at the crane boom *BO*_2_. We assume that the crane boom *BO*_2_ rotates with a constant angular velocity *ω*_*e*_ around vertical axis *DO*_2_. We then transfer the origin of coordinate system *O* *xy* (Figure 1) to the point *A*_dyn_ of dynamic equilibrium for load *M* and assume that *x*_1_ = *x*; *y*_1_ = *y* + *y*_dyn_ (Figure 3). Then, the second equation of system (15) defines the amount of dynamic deflection *y*_dyn_ = *A*_st_ *A*_dyn_ = (*ω*_*e*_^2^ *Rl*)/(*g* − *ω*_*e*_^2^ *l*).

Relative swaying paths of payload *M* in the plane of the introduced relative coordinates *x* and *y* are shown in Figure 3 for successive increasing swaying times *t*_1_ = 1.25 s (Figure 3(a)), *t*_2_ = 3 s (Figure 3(b)), *t*_3_ = 10 s (Figure 3(c)), and *t*_4_ = 25 s (Figure 3(d)).

Then, we have the normal system of two linear homogeneous differential equations of second order for relative motion of the load *M*:

(16) (D2−(ωe2−(gl))−2ωeD2ωeDD2−(ωe2−(gl)))  ×(x(t)y(t))=0, D=ddt.

The determinant of natural frequencies matrix for the system (16) has the following form:

(17) $$\begin{array}{c} \left|\begin{array}{cc} \lambda^{2}+\left(\frac{g}{l}\right)-\omega_{e}^{2} & -2\cdot \omega_{e}\cdot \lambda \\ 2\cdot \omega_{e}\cdot \lambda & \lambda^{2}+\left(\frac{g}{l}\right)-\omega_{e}^{2} \end{array}\right|=0. \end{array}$$

Using (17), we adjust the roots *λ*_1_ and *λ*_2_ of secular equation:

(18) $$\begin{array}{c} \lambda_{1}=\pm \nu_{1}\cdot i,\;\;\lambda_{2}=\pm \nu_{2}\cdot i,\;\;\nu_{1}=k+\omega_{e},\\ \nu_{2}=k-\omega_{e},\;k=\sqrt{\frac{g}{l}},\;\omega_{e}\neq k. \end{array}$$

The initial conditions for the problem are as follows:

(19) $$\begin{array}{c} x\left(0\right)=0,\;\;Dx\left(0\right)=+V_{B}=+\omega_{e}R,\\ y\left(0\right)=-y_{\text{dyn}},\;\;Dy\left(0\right)=0. \end{array}$$

Taking into account (18) and (19), the law of relative motion for payload takes the following form:

(20) $$\begin{array}{c} x\left(t\right)=A_{1}\frac{i}{2}\left(e^{-\nu_{1}it}-e^{\nu_{1}it}\right)+A_{2}\frac{i}{2}\left(e^{-\nu_{2}it}-e^{\nu_{2}it}\right),\\ y\left(t\right)=A_{1}\frac{1}{2}\left(e^{-\nu_{1}it}+e^{\nu_{1}it}\right)-A_{2}\frac{1}{2}\left(e^{-\nu_{2}it}+e^{\nu_{2}it}\right). \end{array}$$

After substitution of values of *A*_1_ and *A*_2_ in (20), we have the following law of payload *M* relative swaying motion with the uniformly rotating pivot center:

(21) x(t)=(VB−ydyn(k−ωe)2k)i2(e−ν1it−eν1it) +(VB+ydyn(k+ωe)2k)i2(e−ν2it−eν2it),y(t)=(VB−ydyn(k−ωe)2k)12(e−ν1it+eν1it) −(VB+ydyn(k+ωe)2k)12(e−ν2it+eν2it).

The relative trajectories for load *M* on the cable in Figure 3 have been derived for the following numerical values: *R* = 0.492 m; *g* = 9.81 m/s^2^; *l* = 0.825 m; *k* = (*g*/*l*)^0,5^ ≈ 3.448 rad/s; *T* = 30 s; *ω*_*e*_ = 2*π*/*T* ≈ 0.209 rad/s; *α*_1dyn_ = 0.00221 rad; *V*_*B*_ = 0.103 m/s; *y*_dyn_ = 0.00182 m; *ν*_1_ = *k* + *ω*_*e*_ = 3.658 rad/s; *ν*_2_ = *k* − *ω*_*e*_ = 3.239 rad/s (Figure 3).

**Figure 4:**
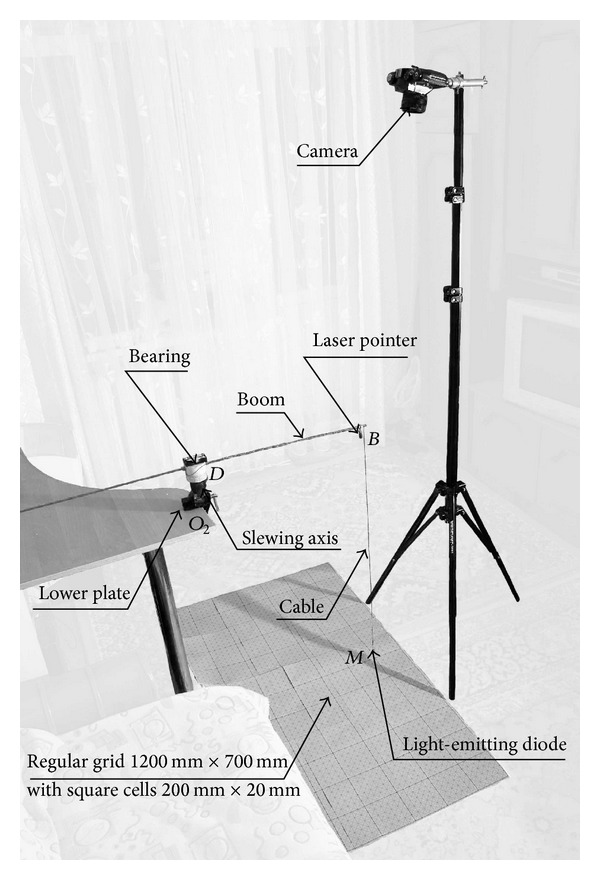
The experimental measurement system for payload *M* swaying during crane boom slewing.

**Figure 5:**
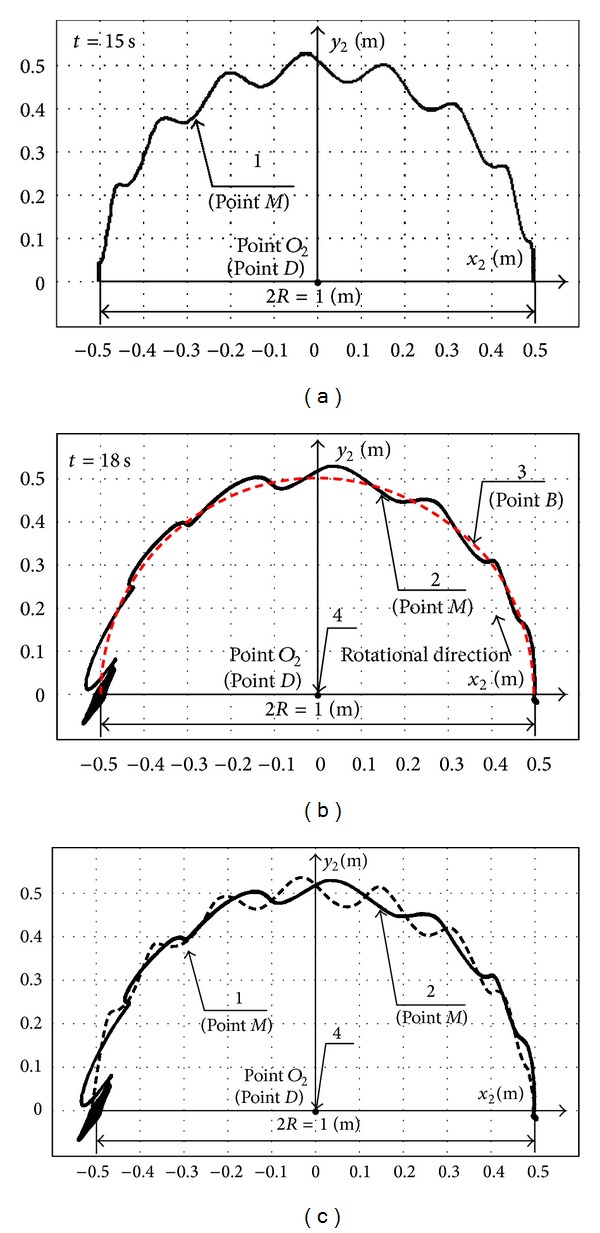
Calculated 1 (a, c) and experimental 2 (b, c) absolute trajectories for load *M*, where 3 is the trajectory of boom point *B* (b); 4 is the center *O*_2_ (*D*) of boom rotation (b, c).

Numerical plots in Figure 3 show the payload *M* swaying in the noninertial reference frame (*O* *xy* *z*). In order to visualize payload *M* absolute swaying path (Figures 4 and 5) in the fixed inertial reference frame (*O*_2_ *x*_2_ *y*_2_ *z*_2_), we will address the following transition formula:

(22) $$\begin{array}{c} \left(\begin{array}{c} x_{2}\left(t\right)\\ y_{2}\left(t\right) \end{array}\right)=\left(\begin{array}{cc} \sin\left(\omega_{e}t\right) & \cos\left(\omega_{e}t\right)\\ -\cos\left(\omega_{e}t\right) & \sin\left(\omega_{e}t\right) \end{array}\right)\left(\begin{array}{c} x\left(t\right)\\ y\left(t\right)+R+y_{\text{dyn}} \end{array}\right). \end{array}$$

Derived computational plot for absolute payload swaying path in the absolute coordinates' plane (*x*_2_ *y*_2_) is shown in Figure 5(a) for load *M* swaying time *t*_1_ = 15 s and crane boom transport slewing angle *φ*_*e*_ = 180°. In Figure 5(b), we show the experimental plot for absolute payload swaying path in the absolute coordinates' plane (*x*_2_ *y*_2_) for load *M* swaying time *t*_2_ = 15 s and crane boom transport slewing angle *φ*_*e*_ = 180°, which was built with an introduction of experimental setup in Figure 4.

## 6. Comparison of Derived and Known Published Results

The computational dimensionless cable force *N*/*mg*, derived in the first approximation and shown in Figure 2, outlines the minor change of the cable tension force in the vicinity of the ordinate (*N*/*mg*) = 1: (*N*/*mg*)∈[0.9875; 1.0233] for *l* = 0.206 m in Figure 2(a); (*N*/*mg*)∈[0.994; 1.0122] for *l* = 0.412 m in Figure 2(b); (*N*/*mg*)∈[0.998; 1.004] for *l* = 0.618 m in Figure 2(c); and (*N*/*mg*)∈[0.99933; 1.0013] for *l* = 0.825 m in Figure 2(d). So the assumption (14) really takes place as shown in Figure 2. The same effect for (*N*/*mg*)_Mitrev_ ∈ [0.94; 1.04] has been derived by Mitrev and Grigorov, [23, Mitrev's Figure 7 in page 89], that confirms assumption (14). An analogous effect for dimensionless cable force (*N*/*mg*)_Maczynski_ ∈ [0.996; 1.005] has been derived by Maczynski and Wojciech, [24, Maczynski's Figure 10 in page 278], which also reaffirms the assumption (14).

In published work by Sakawa et al. ([25], Figure 3 at page 552, Figure 4 at page 552, Figure 5 at page 554, and Figure 6 at page 554), the small Sakawa values *dz*/*dt* ≤ 0.1 (Figures  3 and 4) and *dz*/*dt* ≤ 0.05 (Figures  5 and 6) of *z*-projections for payload velocities have been shown, which confirms the small angle assumption *N* ≈ *mg* (14).

## 7. Discussion and Analysis of Basic Results for Mathematical and Physical Simulation

The relative trajectories for load *M* swaying in Figure 3 show equality of maximum values for relative *x* and *y* payload coordinates:

(23) $$\begin{array}{c} x_{\max}=y_{\max}=A_{1}+A_{2}=\frac{\omega_{e}\left(R+y_{\text{dyn}}\right)}{k}. \end{array}$$

The formula (23) outlines that values *x*_max_ and *y*_max_ in Figure 3 increase with growth of boom slewing velocity *ω*_*e*_ and with increased cable length *l*. The *y*_dyn_ influence on the values of *x*_max_ and *y*_max_ is negligible.

Equation (18) shows that the free oscillation frequencies *ν*_1_ and *ν*_2_ depend on the boom slewing velocity *ω*_*e*_ and natural frequency *k* of the payload with a fixed pivot point *B*. The frequencies *ν*_1_ and *ν*_2_ essentially depend on the pendulum length *l* because *k* ≫ *ω*_*e*_. Kinematically saying, the miscoordination of frequencies *ν*_1_ and *ν*_2_ is *ν*_1_ − *ν*_2_ = 2*ω*_*e*_ and is determined by the crane boom slewing velocity *ω*_*e*_. Dynamically saying, the value of miscoordination is governed by the Coriolis acceleration-induced D'Alembert's inertia force.

Computations shown in Figure 3 for relative payload *M* swaying show that the clockwise direction of rotation for relative oscillations is opposite in direction from the counterclockwise direction of rotation for the crane boom. This effect follows from the conservation of position of the plane *y*_2_ *z*_2_ of oscillations of load *M* in the fixed inertial coordinate system *x*_2_ *y*_2_ *z*_2_.

The comparative analysis of computational (1) and experimental (2) absolute payload trajectories is shown in Figure 5(c). The quantity of the local maximums in Figure 5(a) is 8 for theoretical curve 1. So the theoretical period for relative load swaying is *T*_theor_ = 1.87 s. The quantity of the local maximums in Figure 5(b) is 7.5 for experimental curve 2. The period for experimental curve 2 in Figure 5(b) is *T*_experim_ = 2 s. The discrepancy between the values of theoretical and experimental periods does not exceed 7%.

The experimental absolute trajectory (2) in Figures 5(b) and 5(c) outlines the additional relative swaying of payload *M* after crane boom stop, which has the elliptical form in the vicinity of the point (0, −0.5) in the plane *x*_2_ *y*_2_ for *φ*_*e*_ = 180°. The remaining payload motions after crane boom stop are the small oscillations of the payload with the fixed pivot center.

## 8. Final Conclusions

The closed system of nonlinear ODEs for payload relative swaying has been derived in relative Cartesian coordinates with an introduction of Lagrange equations and a geometric constraint equation.

The explicit form of the dependence of the cable reaction force as a function of the relative payload coordinates *x*_1_, *y*_1_, *z*_1_ and their first and second time derivatives has been found. Derived complex dependency of the cable reaction force on the above mentioned variables determines the nonlinearity of the present problem.

The deviation of cable reaction force from payload gravity force is small. This fact allowed the derivation of a linearized ODE system for payload swaying in relative Cartesian coordinates and verified the small relative Cartesian coordinates' assumption. The analytical expressions for relative payload path have been derived.

Derived results have good agreement with experiments and known published data.
